# Rational use of drugs for the elderly during the COVID-19 pandemic in China: Challenges and opportunities

**DOI:** 10.3389/fphar.2023.1106144

**Published:** 2023-01-20

**Authors:** Qingqing Yin, Dandan Feng, Yong Zhao, Xiaojuan Zhu, Yan Dong, Jianchun Wang

**Affiliations:** ^1^ Department of Geriatrics, Shandong Provincial Hospital Affiliated to Shandong First Medical University, Jinan, Shandong, China; ^2^ Department of Geriatric neurology, Shandong Provincial Hospital Affiliated to Shandong First Medical University, Jinan, Shandong, China; ^3^ Department of Geriatric Hematology and Oncology, Shandong Provincial Hospital Affiliated to Shandong First Medical University, Jinan, Shandong, China; ^4^ Department of Cardiology, Shandong Provincial Hospital, Shandong First Medical University, Jinan, Shandong, China; ^5^ Department of Geriatric Cardiology, Shandong Provincial Hospital Affiliated to Shandong First Medical University, Jinan, Shandong, China

**Keywords:** rational use of drugs, elderly, COVID-19, challenges, opportunities

## 1 Introduction

Since the outbreak of Coronavirus disease 2019 (COVID-19), it has a global pandemic, leading to an unprecedented setback for global public health and economy (Hu et al., 2021). Nowadays, with the popularization of vaccination and the accumulation of experience in epidemic prevention and control, the case fatality rate and mortality rate of COVID-19 have been continuously reduced worldwide. But the global number of new COVID-19 infections has increasing dramatically every day, and this trend is also striking in China. At present, most of the severe or dead COVID-19 patients were elderly people, especially those with chronic underlying diseases (Chen et al., 2021). Due to aging and underlying diseases, the elderly have reduced immunity and complicated conditions, leading to prolonged viral clearance and even death. Therefore, the elderly are the key population for prevention and control of COVID-19.

During the COVID-19 pandemic, in order to avoid cross-infection caused by population aggregation, for elderly people with chronic diseases, hospital visits should be avoided if there is no deterioration of diseases. So it is essential for the elderly to use drugs rationally at home to control the progression of diseases. As China enters the aging society, the prevalence of age-related geriatric and crippling diseases increase gradually. Due to the coexistence of multiple diseases, the decline of organ function and the decrease of self-care ability, there have been various problems for the rational use of drugs in the elderly (Lai et al., 2018). Especially in the case of COVID-19, the rational drug use of the elderly faces greater challenges.

## 2 Problems of rational use of drugs in the elderly

### 2.1 Polypharmacy in the elderly

The elderly usually suffer from a variety of chronic diseases, and needs many kinds of medications, triggering problems of misuse and abuse of drugs (Munger, 2010). The reasons for this include: repeated medication was prescribed by multiple doctors in multiple hospitals; the patients bought non-prescription drugs without authorization; the patient stopped taking the drug without permission after the symptoms improved.

### 2.2 The tolerance to drugs decreases in the elderly

Due to the loss of body mass, especially the decrease of liver and kidney function which are closely related to metabolic and excretory functions, the safe range of medication becomes narrow, and the elderly are prone to drug poisoning. When multiple drugs are taken at the same time and the dosage does not change, adverse drug reactions and drug interactions are likely to occur (Mortazavi et al., 2016).

### 2.3 Medication compliance is worse in the elderly

Loss of memory and attention leads to the elderly missing or forgetting to take medicine, and the elderly usually stop, increase or reduce the dose without strictly following the doctor’s advice, greatly affecting the efficacy of drugs or causing adverse drug events (Sun et al., 2018).

## 3 Challenges of rational drug use in the elderly during the COVID-19 pandemic

The elderly are at high risk of severe case caused by severe acute respiratory syndrome coronavirus 2 (SARS-CoV-2), especially those with underlying diseases, such as cardiovascular and cerebrovascular diseases, respiratory diseases, tumors and chronic renal insufficiency. In order to reduce the infection rate of the virus, the elderly are encouraged to avoid going out or quarantine at home. As a result, the rhythm of life of the elderly is disrupted. The elderly may experience depression associated with reduced outdoor activities and visits from loved ones, which may adversely affect their physical health, even aggravate the condition.

Due to the impact of COVID-19, elderly people with chronic diseases may not be able to go to the hospital for regular review, resulting in delayed medication dose adjustment or delayed treatment in the early stage of the disease. Moreover, many elderly people living alone do not go to the hospital in time for medicine or stop taking medicine by themselves, which affects the control of the disease. Although people have the option of doctor visits online, most elderly people living alone do not know how to operate smartphones and cannot communicate with doctors through wechat or video conferencing (Lv et al., 2021).

## 4 Opportunities and strategies of rational medication in the elderly during the COVID-19 pandemic

Catalyzed by the COVID-19 pandemic, Internet medical services have developed rapidly in China, including health education, medical information query, electronic health records, disease risk assessment, disease consultation online, electronic prescription, remote consultation, remote treatment and rehabilitation and other forms of healthcare services using the Internet as a carrier and technical means. Family members or community volunteers should encourage and assist the elderly to use the electronic platform to communicate frequently and timely with community doctors and specialists, so that they can grasp the health status of the elderly in time and establish a good communication platform and bridge between doctors and patients, so as to regularly assess the condition and adjust treatment strategies.

During the COVID-19 epidemic, the offspring should strengthen their love and care for the elderly, advise the elderly to develop a healthy lifestyle, carefully observe their parents’ changes of physical condition, help their parents formulate and implement chronic disease management, and conduct regular comprehensive physical assessment. For the elderly who live alone, family members or community volunteers had better help them to establish a medication card indicating the time and dose of medication to be taken, and often urge the elderly to take medicine according to the doctor’s advice on time and standard, and do not stop or reduce medicine without permission.

The elderly should be sure to use drugs regularly according to the plan formulated by the doctor, and should not reduce or stop the drug because the remaining drugs are not enough. Patients or family members can consider going to community hospitals and formal retail pharmacies near their homes to renew prescriptions, or purchase enough drugs through pharmacies or qualified online channels for a full course of treatment. If the aged have physical discomfort, they should inform their families, community service personnel or nursing home staff in time. The elderly should also develop a good habit of monitoring their body temperature, blood pressure and heart rate every day, keep simple and proper exercise, pay attention to a reasonable diet, quit smoking and limit alcohol, and keep a good mood.
